# Incremental analysis of the reengineering of an outpatient billing process: an empirical study in a public hospital

**DOI:** 10.1186/1472-6963-13-215

**Published:** 2013-06-13

**Authors:** Kuan-Yu Chu, Chunmin Huang

**Affiliations:** 1 Quality Management Group of Administrative Center, Tao-Yuan General Hospital, Taoyuan, Taiwan; 2 Cost Accounting Section, Department of Accounting, Taipei Veterans General Hospital, Taipei City, Taiwan

**Keywords:** Time and motion study, Sensitivity test, Incremental analysis, Smartcard, Reengineering

## Abstract

**Background:**

A smartcard is an integrated circuit card that provides identification, authentication, data storage, and application processing. Among other functions, smartcards can serve as credit and ATM cards and can be used to pay various invoices using a ‘reader’. This study looks at the unit cost and activity time of both a traditional cash billing service and a newly introduced smartcard billing service in an outpatient department in a hospital in Taipei, Taiwan.

**Methods:**

The activity time required in using the cash billing service was determined via a time and motion study. A cost analysis was used to compare the unit costs of the two services. A sensitivity analysis was also performed to determine the effect of smartcard use and number of cashier windows on incremental cost and waiting time.

**Results:**

Overall, the smartcard system had a higher unit cost because of the additional service fees and business tax, but it reduced patient waiting time by at least 8 minutes. Thus, it is a convenient service for patients. In addition, if half of all outpatients used smartcards to pay their invoices, along with four cashier windows for cash payments, then the waiting time of cash service users could be reduced by approximately 3 minutes and the incremental cost would be close to breaking even (even though it has a higher overall unit cost that the traditional service).

**Conclusions:**

Traditional cash billing services are time consuming and require patients to carry large sums of money. Smartcard services enable patients to pay their bill immediately in the outpatient clinic and offer greater security and convenience. The idle time of nurses could also be reduced as they help to process smartcard payments. A reduction in idle time reduces hospital costs. However, the cost of the smartcard service is higher than the cash service and, as such, hospital administrators must weigh the costs and benefits of introducing a smartcard service. In addition to the obvious benefits of the smartcard service, there is also scope to extend its use in a hospital setting to include the notification of patient arrival and use in other departments.

## Background

Traditional cash billing services require that a patient leaving an outpatient clinic go to the cashier window, take a numbered ticket, and wait for their number to be called before seeing a clerk for payment. They then pay their bill using cash and receive any change owed to them. The patient is then free to go to the pharmacy. However, in recent times the satisfaction levels of patients have been affected to a greater degree by the waiting time and convenience level of the cash system [1,2]. Thus, outpatient department (OPD) billing processes in various hospitals have been reengineered [3].

Reengineering is the radical redesign of a system process to achieve dramatic improvements in cost, service, and speed [4]. Previous studies have used hospital incremental cost analyses to determine the cost of specific medical services [5,6], and then compared these with alternative medical procedures [7,8]. Appropriate strategies can then be identified to minimize costs and maintain efficient medical services [9,10].

The most common smartcard in Taipei, named EasyCard, uses radio-frequency identification (RFID) technology and is a contactless smartcard system. It has been used for payment on the Taipei MRT, buses, and other public transport services since June 2002. Its use has now been expanded to include convenience stores, department stores, supermarkets, and hospitals [11].

Smartcard systems are becoming more common in hospitals. For example, smartcard billing services in hospitals enable outpatients to use their smartcards to pay for their procedures. In contrast with the traditional cash system, the smartcard service does not require any waiting in line. The patient is asked by the attending nurse whether they have a smartcard. If so, then the card is taken by the nurse and placed in the card reader and the card balance will show. If there are adequate funds available then the cost of the hospital visit is deducted from the card’s balance, the patient receives a receipt, and can proceed to the pharmacy to fill their prescription.

In this study, we use a time and motion study to determine the time it takes to pay an invoice in both systems [12-14], and a cost analysis and cost-volume-profit analysis to determine the unit cost and the incremental cost of the systems [15,16]. A sensitivity analysis is also performed to determine the optimal mix of smartcard use and number of cashier windows to produce the lowest incremental cost and the greatest reduction in waiting time.

There are a number of costs associated with the two procedures dealt with in this study. We aim to measure the unit cost (the unit cost is the cost incurred by a company to produce, store and sell one unit of a particular product, and includes all fixed and variable costs), and use the following definitions: the unit cost includes (i) direct costs, such as labor, materials, depreciation, and repair costs; and (ii) indirect costs, such as services (utilities: power, fuel, electricity), administrative costs, and research and training (R&T) costs. Other costs include patent royalties (smartcard service fees) and business tax.

Direct labor and material costs can be traced to particular cost objects. Idle time, which is defined as the unproductive time spent by employees due to factors beyond their control, has also been considered in previous studies [17]. Idle time is very important to reduce the costs of labor because an increase in idle time will increase production costs [18]. Indirect costs are traced to the cost object using a cost driver, which is a measure of an activity. The time spent on an activity is used in principle as a cost driver [19]. Time and motion studies have been used to compute the time spent on various activities.

A new billing service would represent a significant change in a hospital’s process, and as such, a hospital healthcare manager would have to fully understand the costs and benefits of the new billing service to effectively allocate resources. The aims of this study are as follows: (1) to measure the waiting time and process time for two billing services—the new smartcard service and the traditional cash procedure, (2) to measure the costs for each procedure, and (3) to perform a sensitivity test of incremental cost and waiting time.

## Methods

The study was conducted in the New Taipei City Hospital, a public teaching hospital located in New Taipei City, Taiwan. This hospital saw approximately 28,800 outpatients over the one-month study period. A smartcard billing service system was installed in the hospital OPD in July 2008.

### Implementation procedures

**Figure 1:**
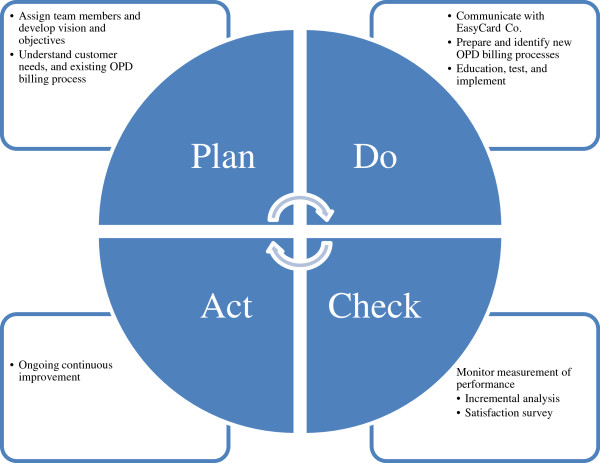
PDCA for the implementation of the reengineered smartcard billing system.

PDCA (plan–do–check–act), the Deming cycle, is an integrative four-step management method used in organizations for the control and continuous improvement of processes and products/services [20]. Figure 1 details the PDCA method used by the hospital to implement the new smartcard billing system. First, the hospital planned the move (‘Plan’); they assigned team members and developed their vision and objectives. They then sought to identify customer needs and the existing OPD billing process. Second, under the ‘Do’ aspect, the hospital communicated with EasyCard Co., and designed the new OPD billing processes. This was followed by various education sessions, testing, and the implementation of the system. Next came the ‘Check’ process, whereby the hospital monitored the performance of the system, for example, performing an incremental analysis and satisfaction survey. The final step is ‘Act’, which involves ongoing improvements.

The study period for this study was July 2008 to June 2009. By analyzing outpatients’ billing activities and the financial statements of the hospital, we were able to determine the length of time spent waiting, paying hospital invoices by cash/smartcard, and the cost of these activities. We were also able to estimate the unit costs of the hospital cash billing service and the smartcard billing service, and conduct an incremental analysis. A sensitivity test was then performed to predict alterative incremental costs and waiting time. Ethical clearance was issued by the Ethics Committee of the New Taipei City Hospital.

### Data collection

#### Payment time measurement

**Figure 2:**
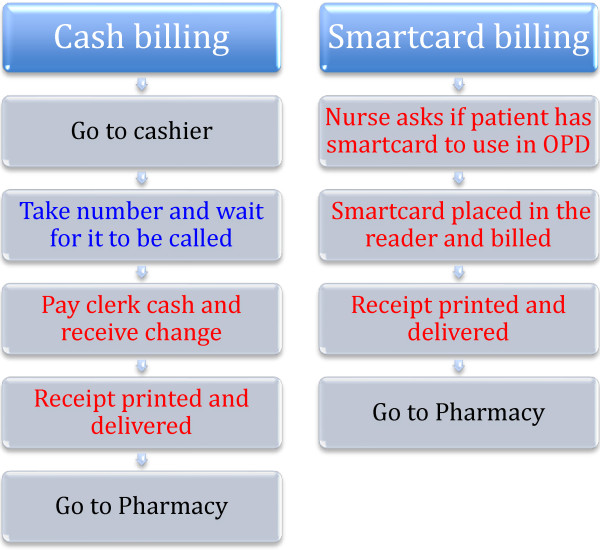
Billing process: blue - waiting time; red - activity time.

This study compared the time required to pay hospital invoices via the new billing system and the traditional cash system. To estimate the activity time required for each process, a task analysis of both the cash billing service and smartcard billing service was performed. First, we reviewed the standards of procedures and manuals for each billing service to determine the processes and some of the required materials (Figure 2).

Second, a time and motion study was undertaken to estimate the waiting time involved for the cash billing service and the activity time required for both billing services. Thus, we measured the time patients spent in the cash billing process during 60 periods. We studied two cashier windows from Monday to Friday and one window on Saturdays over a 2-week period: weekday mornings (10:00 and 11:00) and afternoons (14:30 and 15:30) and two Monday and Thursday evening sessions (19:00 and 20:00) and Saturday (10:00 and 11:00) in the first and third week in May 2009. Each period recorded ten cases. The ‘waiting time’ began when the patient took a number and ended when the number was called. The ‘activity time’ for the cash billing service is defined from when the patient stood before the clerk to pay the bill to the printing of the receipt and its delivery. The ‘activity time’ of the smartcard billing service is from when the smartcard is placed in the reader to the printing of the receipt and its delivery.

#### Unit cost assessment

We examined the hospital balance sheets and income statements (included in the 2008 end of year report) to estimate direct costs (prices that can be completely attributed to services). These include labor (service labor costs and supporting labor costs), materials, depreciation, and repair costs. Indirect costs (costs that are not directly accountable to a cost object) were also identified: services (utilities including power, water, fuel) administrative costs (e.g., accounting, contracting, and industrial relations), R&T, and other costs (patent royalties and taxes) using the account classification method. Table 1 shows the three cost groups, their operational definitions of unit cost, and their subsequent cost. The unit cost for both alternatives were evaluated.

**Table 1:** Billing cost items and their operational definitions (NT$)

**Cost items**	**Operational definition**	**Cost**
**Direct costs**		
**Labor**	Cost × Quantity ÷ (1- idle time%) × T*	Cost/min: nurse 5.49; clerk 4.68; Idle time: 20%; T = activity time ÷ working time
**Material**	Cost × Quantity	
**Depreciation**		
Buildings/construction	Cost of building ÷ depreciation life × T	Cost of building/m^2^ = 21,749 Depreciation life = 50 yrs
Machinery/equipment	Cost ÷ Depreciation life × T	
**Repair and maintenance**		
Buildings/construction	Cost of building repair × area × T	Repair cost of building/m^2^ = $59.14 (cash billing area = 18.5 m^2^; smartcard area = 13.8 m^2)^
Machinery/equipment	Cost × equipment repair% × T	Equipment repair% = 2.42%
**Indirect costs**		
Service	Direct cost ÷ (1-Service%) × Service% × T	Service% = 11.23%
Administration	(Direct cost + Service costs) × Administration% × T	Administration% = 3.58%
Research & training	(Direct cost + Service costs) × R&T% × T	R&T% = 2.36%
**Other costs**		
Patent royalty	Amount × 1.5%	
Business tax	Amount × 0.05%	

Working time = 132 working hours per month × 60 min × 12 months.

#### Prediction of incremental cost and waiting time

We used OPD volume, percentage of smartcard billing, and unit cost cash billing to predict OPD billing cost. The incremental cost was the difference between the original and reengineered billing systems. The waiting time of the cash billing was estimated using original waiting times and clerk loading after reengineering (Table 2).

**Table 2:** Incremental cost and waiting time of reengineered billing systems

**(O) Origin**	**(R) Reengineering**	**Incremental cost**	**Waiting time**
OPD Volume × unit cost of cash billing	Volume cash billing × unit cost of cash billing + volume smartcard billing × unit cost of smartcard billing – clerk compensation saving	(R) − (O)	Original cash billing waiting time × clerk loading

### Data analysis

To estimate the unit cost of the cash and smartcard billing services, we designed a formula using Microsoft Office Excel software. We calculated the unit cost after inputting the cost data. A cost comparison was performed, comparing the total cost for each payment system.

A sensitivity analysis was also performed, looking at the effect of smartcard use and number of clerks on the incremental cost and estimated waiting time.

We conducted a sensitivity analysis based on the following assumptions.Assumption 1:: number of patients at OPD is fixed.; Assumption 2:: billing speed of clerks is equal. 

We used the following independent variables: number of OPD patients: 28800; Billing team: 5 clerks.

## Results

### Activity time

We used a time and motion study to identify the different sample groups in our study: we identified 60 sample groups to determine the activity time of both billing services, including 32 cash billing sample groups to determine the waiting time of the cash billing service. The average activity time for the cash billing service was 0.93 min, with an average waiting time of 8.05 min. The average activity time of the smartcard billing service was 0.75 min. There was no waiting time for smartcard users as patients paid their bill using the smartcard before they left the examination room.

### Historical data

According to the data sourced from the hospital’s financial records, the unit cost is made up of the following (see Table 1): (i) labor cost/min: nurse $5.49, clerk $4.68, idle time 20%; (ii) cost of building/m^2^ = $21,749, depreciation life of the building was set at 50 years, unit area of cash billing services = 18.5 m^2^, unit area of smartcard billing services = 13.8 m^2^; (iii) Working time = 132 working hours per month × 60 min × 12 months; (iv) repair costs of building/m^2^ = $59.14; equipment repairs% = 2.42%; (v) services% = 11.23%, administration% = 3.58%, R&T = 2.36%. In addition, the average unit cost of smartcard service fees and business tax was $2.87 from July 2008 to June 2009. Table 3 indicates the unit cost of direct labor and materials in both billing services. Table 4 shows the depreciation and repair costs for both services. Please note that where the depreciation differs for the same piece of equipment under each service it is because that item is used either more or less than under the alternative system. For example, the server is used less under the smartcard system and so its level of depreciation is less.

**Table 3:** Unit cost of direct labor and materials in cash and smartcard billing services

	**Labor**					**Material**			
	**Item**	**Rate**	**Quantity**	**Activity time (min)**	**Amount**	**Item**	**Cost**	**Quantity**	**Amount**
Cash billing	Cashier clerk	4.68	1	0.93	4.35	Thermal paper (receipt paper)	130	0.001	0.13
						Pre-inked stamp	100	0.001	0.01
Smartcard billing	Nurse in OPD	5.49	1	0.75	4.12	Thermal paper	130	0.001	0.13
						Pre-inked stamp	100	0.001	0.01

**Table 4:** Depreciation and repair costs for cash and smartcard billing services

		**Cash billing**				**Smartcard billing**			
	**Cost items**	**Cost**	**Depreciation life (yr)**	**Activity time (min)**	**Amount (NT$)**	**Cost**	**Depreciation life (yrs)**	**Activity time (min)**	**Amount (NT$)**
Equipment	Server	107,242	5	0.93	0.21	107,242	5	0.75	0.17
	Electronic draft capture					23,183	5	0.75	0.04
	Expansion card	680	5	0.93	0	680	5	0.75	0
	Network cable	90	5	0.93	0	90	5	0.75	0
	Screen	12,000	5	0.93	0.02	12,000	5	0.75	0.02
	Keyboard	210	5	0.93	0	210	5	0.75	0
	Printer	8,600	5	0.93	0.01	8,600	5	0.75	0.01
	Numbering system	9,000	5	0.93	0.02				
	Chairs	1,050	5	8.05	0.02				
	Card reader	2,600	5	0.93	0.01				
Sum					0.29				0.24
Building			4.18 m^2^	8.05	0.51		5.60 m^2^	0.75	0.06
Repair					0.11				0.05
Total					0.92				0.35

### Unit cost

Table 5 shows the total unit cost for the cash billing service is $6.47, and $8.41 for the smartcard billing service. The reason for smartcard service having a higher unit cost is that the smart card was also subject to service fees and business tax. The unit incremental cost was $1.94. In this case, the cost-benefit of the smartcard billing services in this hospital was negative.

**Table 5:** Unit cost (NT$) of cash billing and smartcard billing

	**Cash billing**	**Smartcard billing**
**Cost items**	**Amount (%)**	**Amount (%)**
**Labor**	4.35 (67.2)	4.12(74.4)
**Material**	0.23 (3.6)	0.23 (4.1)
**Depreciation & Repair**	0.91 (14.1)	0.35 (6.3)
**Indirect**	0.98 (15.1)	0.84(15.2)
**Sum**	6.47	5.54
**Other**		2.87
**Total**	6.47	8.41

### Cost effectiveness

The results show that the smartcard billing system is quicker than the traditional billing system with no waiting in line and a short transaction period (compared with queuing and dealing with a cashier and cash in the traditional system). However, the traditional system has a lower unit cost than the new system.

### Sensitivity analysis

Our results showed that if half of all OPD patients used smartcards to pay their invoices and there were four cashier windows in operation, then waiting time would be reduced from 8.05 min to 5.03 and the hospital saves NT$1,697, which is close to breaking even (Table 6).

**Table 6:** Sensitivity analysis of effect of smartcard use and cashier windows on incremental cost and waiting time

**Number clerks**	**% Smartcard**	**Volume cash**	**Volume smartcard**	**Reengineering billing cost**	**Incremental cost**	**Waiting time estimation**	**Time saving**
5	0.0%	28800	0	186,336	0	8.05	0
	10.0%	25920	2880	191,923	5,587	7.25	0.81
	20.0%	23040	5760	197,510	11,174	6.44	1.61
	30.0%	20160	8640	203,098	16,762	5.64	2.42
	40.0%	17280	11520	208,685	22,349	4.83	3.22
	50.0%	14400	14400	214,272	27,936	4.03	4.03
4	0.0%	28800	0	156,703	−29,633	10.06	−2.01
	10.0%	25920	2880	162,290	−24,046	9.06	−1.01
	20.0%	23040	5760	167,877	−18,459	8.05	0
	30.0%	20160	8640	173,465	−12,871	7.04	1.01
	40.0%	17280	11520	179,052	−7,284	6.04	2.01
	50.0%	14400	14400	184,639	−1,697	5.03	3.02
	53.0%	13536	15264	186,315	−21	4.73	3.32

## Discussion

To measure the performance of smartcard billing, this study focused on the incremental cost of reengineering the billing system and the waiting time for cash billing. In using the smartcard service, patients reduced their waiting time by at a maximum of 8 minutes (as they did not have line up to see a cashier), and could immediately proceed to the hospital pharmacy to fill their prescription. The results reflect the benefits of the smartcard procedure. Although the waiting time was reduced (and users did not have to carry large amounts of cash with them), the unit cost of the smartcard billing service was higher than that of the cash billing service. Thus, hospitals have to weigh the unit cost against the obvious benefits. However, it is possible that the unit cost will decrease overtime as the hospital and patients better understand the new system. The sensitivity analysis showed that the hospital would be close to breaking even if 50% of patients used smartcards to pay their invoice, with four clerks in operation.

While the smartcard billing service had a larger unit cost than the traditional cash service, the difference can be considered relatively minor, and could be justified by taking into account the decrease in time and increased convenience. It can also be inferred that the hospital successfully created a positive image by providing patients with convenient service. Moreover, users were likely to have enjoyed the benefits of convenience, time saving, and the extra security that the card provided (such as not needing to worry about carrying large amounts of cash).

Furthermore, hospitals are non-profit institutions with patient-centered ethics, and should strive to make a patient’s time in the hospital as smooth as possible. Public hospitals have a key mission to provide community service, and this is only furthered by the availability of a smartcard billing service.

We found that the main difference between the cash billing service and smartcard billing service was the waiting time involved in cash billing and the giving of change. Users of the smartcard service saved at least 8 minutes by not having to use the cashier window and they did not have to carry a large amount of cash for outpatient transactions.

Such a system could also be used elsewhere in the hospital. For example, it could be implemented in other departments such pharmacies and in radiology departments that are frequently used by outpatients. If the system was used on a larger scale it could increase facility productivity and efficiency. Furthermore, the system could also be used by patients (i.e., swiping their cards) to notify nurses/doctors that they have arrived for their appointment.

From the viewpoint of the cost structure of the smartcard service, the labor cost is the main component of the unit cost. Regarding this hospital, the reengineering of the cash billing service does not increase labor costs. Under a cash billing system clerks are required to work in the cashier windows. With the smartcard service, some billing labor loading referred to nurses in OPD clinics, and thus the hospital would save approximately NT$29,633 per month per clerk. The results of the sensitivity analysis showed that the hospital could save NT$1,697 by removing one clerk, and patients using the cash billing service would save approximately 3 minutes in waiting time. Consequently, the idle time of nurses could also decrease as they would be responsible for processing smartcard payments. In other words, the reengineering of the billing service would improve OPD nurse productivity.

A key consideration is, however, that it is important to reduce the cost of labor because when idle time increases, production costs increase. Idle time is the unproductive time in a production process due to a number of reasons. When increasing production, hospitals must be able to keep track of idle time, and use that information to calculate productivity rates. Based on that information, the hospital can then eliminate idle time. It can be assumed though that idle time may increase as nurses become more proficient with the system.

The cash billing service also has other potential functions: the system could be used for new patient registration and inpatient billing services, and for outpatient billing services when the fees are especially high. Therefore, smartcard billing services cannot, as yet, completely replace cash services. Thus, a cash billing service has the characteristics of a fixed cost, and where cash billing has been replaced by a smartcard service, a part of the cost of cash billing services can be classified a sunk cost (e.g., depreciation, and costs that have already been incurred and cannot be recovered) [21-23]. As the activity level increases, total fixed costs do not change, but the unit fixed cost declines. Thus, the cost analysis focuses on the total fixed cost rather than the fixed cost per unit [24]. Regarding decision-making, it is wise to include a fixed cost in the total cost, rather than as a per-unit cost. Where a fixed cost has been allocated [25], it is then necessary to identify whether or not it is avoidable [26].

The sensitivity analysis shows that we could suppose that hospital administrators install further cash billing windows to reduce the waiting time of cash billing users. While 53% of outpatients used the smartcard billing services, users of the cash billing service could reduce their waiting time by one third. Incremental costs could break-even because of the decrease in direct labor required for cash billing services in the hospital.

It is also important to provide further incentives for people to use smartcard billing services: greater convenience (including increasing the number of smartcard top-up stations in the hospital, special windows in pharmacies for smartcard users, to be used for inpatient services as well) and lower costs (for example, a discount if the patient pays by smartcard). These incentives are likely to be significant factors for users.

Furthermore, in the event that a smartcard transaction is unsuccessful, that is, the funds available on the card are insufficient, patients would add value to their cards at a top-up station or go and visit a cashier window to pay for their visit. If the rate of such crossover was reduced, the total cost for smartcards could be less than that for cash. Such failures would be likely to decrease as patients grow more accustomed to the procedure and remember to keep their card values high. However, in Taiwan, there are very generous government subsidies for hospital treatments and the amounts that patients have to pay are relatively small. These are generally easily paid with pre-paid smartcards, and patients seldom encounter problems paying by smartcard.

## Conclusions

There are some limitations in this study. Different hospitals have different cost behaviors and cost structures and as data were collected from a single hospital this could limit the generalization of the results. Further limitations are also caused by the estimation of costs using historical data and may include information loss, costs not matched to an information period, and the impact of inflation (during periods of inflation, historical cost may not reflect future cost behavior). In addition, the unit of time for which dependent and independent variables were measured may not match.

The new smartcard billing service introduced in the sample hospital presented some drawbacks in terms of cost. However, users saved time and did not have to carry large amounts of cash for outpatient transactions. In addition, we found that labor costs would decrease and productivity would increase under a smartcard system. At the same time, the hospital successfully established an image as a hospital that provides convenient services to patients. To increase the percentage of smartcard billing service users, we suggest that hospital administrators to offer greater incentives to reinforce patronage.

## Competing interests

The authors declare that they have no competing interests.

## Authors’ contributions

KYC constructed the study protocol, and performed the data collection and the statistical analyses. CMH designed the formula for the cost analysis and interpreted the study. Both authors revised, read, and approved the final manuscript.

## Pre-publication history

The pre-publication history for this paper can be accessed here:

http://www.biomedcentral.com/1472-6963/13/215/prepub
